# Stability of Recombinant Tissue Plasminogen Activator at −30 °C Over One Year

**DOI:** 10.3390/ph6010025

**Published:** 2012-12-31

**Authors:** Abdulmalik Alkatheri

**Affiliations:** 1 College of Pharmacy, King Saud bin Abdulaziz University for Health Sciences, Riyadh 11426, Saudi Arabia; 2 National Guard Health Affairs, King Abdulaziz Medical City, Riyadh, 11426, Saudi Arabia; E-Mails: alkatheria@ksau-hs.edu.sa; katheria@ngha.med.sa; Tel.: +966-1-801-1111 (ext. 51006)

**Keywords:** cryopreservation, fibrinolysis, stability, tissue plasminogen activator.

## Abstract

Recombinant tissue plasminogen activator (rt-PA) is used to restore patency and avoid inadvertent removal of peripheral and central venous catheters. rt-PA was reconstituted (1 mg/mL) then cryopreserved at −30 °C for 1, 2, 3, 6, 8, and 12 months and, then its stability was determined. After cryopreservation for one and two months, rt-PA kept more than 95% of its activity compared to standard samples, while cryopreservation for three months caused 8% loss of activity. However, after cryopreservation for six months or more, rt-PA retained only 87.5% or less activity compared to standard samples. Therefore, it is recommended that reconstituted rt-PA be cryopreserved at −30 °C for a maximum period of three months.

## 1. Introduction

Urokinase, streptokinase, and tissue plasminogen activator (t-PA) are fibrinolytic agents that activate plasminogen to form plasmin [1] which dissolves cross-linked fibrin in blood clots and also prevents fibrinogen from forming more fibrin [2,3,4]. rt-PA is a recombinant tissue plasminogen activator that is commercially manufactured by recombinant DNA techniques [5,6].

The use of central venous catheters, as a permanent access in chronic haemodialysis patients, is complicated by clotting [7], which is the major cause of catheter dysfunction [8]. This problem is usually handled by the use thrombolyitc agents, invasive de-clotting procedures or catheter replacement [9]. The aforementioned fibrinolytic agents have been used to lyse clots in order to restore patency and avoid removal of catheters [9]. Traditionally, urokinase has been used as a thrombolytic agent for de-clotting haemodialysis vascular access catheters [5,10,11,12]. Some comparative studies on thromobsed central venous catheters showed that rt-PA is twice as effective as urokinase (*P<0.01*) [13]. In addition, rt-PA was used successfully to regain catheter function when declotting by urokinase failed [14]. It has been recently established that using rt-PA once weekly instead heparin three times a week to de-clot central venous catheters can significantly reduce catheter malfunction and bacteremia [8].

Currently, rt-PA is supplied to institutions in 50 mg vials that are drawn up into 1 mg/mL aliquots that are stored at −30 °C until administration. Hence, only facilities that have access to both a pharmacist and −30 °C freezers will be able to cost-effectively use rt-PA. It has been established that cryopreserved rt-PA at −30 °C for up to three months, is safe and effective (97% remaining activity) for the restoration of the patency of occluded central venous access devices in pediatric oncology patients [15]. It is worth mentioning that the manufactures’ recommendation for rt-PA stability upon reconstitution is eight hours [16]. The stability of cryopreserved rt-PA at −30 °C was assayed over a period of 22 weeks and exceptionally steady rt-PA stability was shown over the entire experimental period [11]. Hence, it would be very interesting to see if this stability trend can be generalized and if it continues beyond the 22 weeks period.

Herein we report the stability of cryopreserved rt-PA at −30 °C over an extended period of one year. The time intervals in this report were 1, 2, 3, 6, 8, and 12 months as the period between three and six month was already covered by the aforementioned study. This study will not only allow for better understanding of the stability profile of cryopreserved rt-PA, it will also help institutions; especially with financial constraints, to establish a cost-effective expiry period for the use rt-PA if it is proven to be stable beyond 22 weeks.

## 2. Results and Discussion

**Figure 1:**
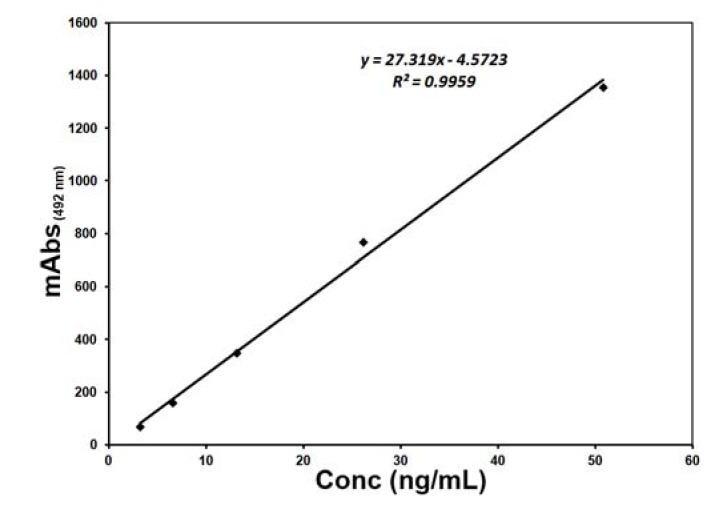
Standard curve for the quantification of cryopreserved rt-PA at −30 °C for 1 month.

Figure 1 shows the standard curve that was constructed for the quantification of rt-PA concentrations after one month of cryopreservation by plotting the milli-absorbance at 492 nm (mAbs_(492 nm)_) versus the concentration in ng/mL.

Results of this assay are shown in Table 1. Regarding cryopreservation for one or two months at −30 °C, the mean concentration of rt-PA in the test samples (49.7 ± 0.1 and 48.1 ± 0.9 ng/mL after one and two months, respectively) retained 99.9 and 96.8% of activity compared to standard samples (49.7 ± 0.1 ng/mL) which was found to be not statistically different when these concentrations were compared using the paired Student’s t-test (*P > 0.05*). After cryopreservation at −30 °C for three months, rt-PA retained 92.0% of its activity (45.7 ± 0.6 ng/mL) which was not statistically different from standard sample, *P > 0.05*. It is clear that there is a significant gradual loss of activity in the cryopreserved samples as one way-ANOVA analysis of the inter-month variability should that the differences between these first three months was statistically significant, *P < 0.05*.

**Table 1:** Effect of freezing for 1 month and 2, 3, 6, 8 and 12 months at −30 °C on the stability of rt-PA.

Sample	rt-PA Concentration (ng/mL)			Mean ± SEM	% Activity
	1	2	3		
**Standard**	49.8	49.6	49.7	49.7 ± 0.1	
**1 month**	49.5	49.6	49.9	49.7 ± 0.2	99.9
**2 months**	48.1	47.2	49.0	48.1 ± 0.9	96.8
**3 months**	-	46.1	45.3	45.7 ± 0.6	92.0
**6 months**	42.6	45.3	43.4	43.8 ± 1.4	88.0*
**8 months**	41.8	43.0	42.8	42.5 ± 0.6	85.5*
**12 months**	38.8	39.1	37.6	38.5 ± 0.8	77.5*

* *P* < 0.05 which is statistically significant, paired t-test.

This trend continues as the bioactivity of rt-PA fell below 90% when the samples were frozen for six months or more at −30 °C. As seen in Table 1, after cryopreservation for 6 months, the mean rt-PA test samples retained 88.0% of activity (43.8 ± 1.4 ng/mL) which is significantly different than that of rt-PA standard samples, *P* < 0.05. Similar results were observed for the samples which were frozen for 8 and 12 months at −30 °C, respectively. The rt-PA test samples retained 85.5% (42.5 ± 0.6 ng/mL) and 77.5% (38.5 ± 0.8 ng/mL) of their activity after 8 and 12 months respectively, which are significantly different than those in the rt-PA standard samples, *P* < 0.05. Again one way-ANOVA analysis showed that the inter-month differences between these concentrations are significant, *P < 0.05*. Lastly, it must be mentioned that all the samples that were sent for microbiological investigation showed no bacterial or candidal growth.

Patients with end stage renal disease use hemodialysis catheters for either temporary or permanent blood access. Recurrent thrombosis and fibrin sheath formation are common causes of poor or inadequate blood flow rates, which require intervention. To resolve this issue, rt-PA has been used to restore occluded catheters. Herein, rt-PA was chosen as the study drug because of its effectiveness as a thrombolytic agent [5,8,12,15]. Although it is expensive, its cost is significantly outweighed by its low effective dose (1 mg/mL), which can be prepared and then frozen, under proper conditions, until used.

Our results are specially important because it showed the gradual loss of stability of rt-PA following freezing at −30 °C for 1-12 months. These results are not in total agreement of those reported by Wiernikowski *et al*. [11]. The researchers in that study have revealed that the stability of rt-PA remains steady under similar conditions for a duration of 22 weeks. Actually, the results of the current study are in line with the recommendation of the National Association of Vascular Access Network, which states that rt-PA, may be stored at −20 °C for at least one month [17]. The trend of gradual loss of activity continued as the cryopreservation of rt-PA at −30 °C was extended to 6 months or more, leading a significant decrease to less than 90%. These results are particularly interesting and highly important since, as mentioned earlier, Wiernikowski *et al*. reported that rt-PA was stable for up to 22 weeks under the same conditions. Furthermore, Calis and his group found that the bioactivity of rt-PA, when stored for 6 months at −20 °C, was indistinguishable in comparison with freshly prepared samples [18]. Our results contradict those of Calis *et al*., as they clearly show that rt-PA retained only 88.0% of the activity after freezing for 6 months. These variations in results might be due to subtle differences in the cryopreservation setting between institutions and laboratories. At the same time, these results do not contradict Shaw *et al*.’s finding, which showed that the enzymatic activity of rt-PA when stored in cryogenic vials at −80 °C for seven years was not decreased [19], since there is a large difference in the temperatures used for the two investigations. These current findings imply that rt-PA can be safely aliquotted into 1 mg/mL syringes and frozen at −30 °C with a generalized stability for a maximum of 3 months, and hence we do not recommend the use of frozen rt-PA beyond this period of storage.

## 3. Experimental

### 3.1. Materials

Fine chemicals were obtained from Sigma-Aldrich Chemical Company (St. Louis, MO, USA; www.sigmaaldrich.com) and ACROS Chemicals (Geel, Belgium; www.acros.com). Bulk solvents were obtained from local vendors. Double distilled water or sterile water for injection were used wherever appropriate. Commercial 50 mg rt-PA was obtained from Genentech Inc (South San Francisco, CA, USA)

### 3.2. Assessment of Stability

For the reconstitution of the commercial 50 mg rt-PA a modification of the Wiernikowski *et al.* procedure was used [11]. First, 50 mg vials were reconstituted with sterile water to yield 50 solutions of 1 mg/mL. The samples were then capped with standard rubber syringe caps, labeled, and separated into plastic freezer bags and stored at −30 °C within one hour of preparation. It is worth mentioning that baseline samples were assayed for rt-PA stability at the first day of reconstitution. Storage in the freezer was maintained for 1, 2, 3, 6, 8 and 12 months and at each time interval, samples were assayed for stability in triplicate.

To prepare samples for the assay, the frozen rt-PA (1 mg/mL) were removed from the freezer and thawed at room temperature until the solution was free of visible crystals. The stability of each sample, as determined by fibrinolytic activity, was assayed using commercially available kits (Spectrolyse® [fibrin] t-PA, Biopool AB, Umea, Sweden) according to the Mataga *et al*. procedure [20]. The fibrinolytic activity of the stored samples was compared with that of standard reference amounts of rt-PA.

It is worth mentioning that a standard curve for the quantification of rt-PA concentration was prepared for each time interval at the time of analysis to guarantee accuracy of results and reduce errors. The curves were established using the materials provided with the assay kit by the manufacturers by plotting the milli-absorbance_(492 nm)_ (mAbs_(492 nm)_) vs. the concentration of rt-PA in ng/mL.

To assay for microbiological contamination, an initial sample at the time of reconstitution, in addition to six samples (1 mg/mL), which were stored at different intervals, were sent for microbiological testing. The samples were incubated at room temperature in tryptic soy broth in thioglycolate. These incubation conditions promote the growth of both aerobic and anaerobic pathogens such as *Staphylococcus epidermidis* and yeast such as *Candida albicans*, which are commonly found in I.V. admixture rooms [7].

To confirm the specificity of the assay, control solutions of rt-PA were subjected to one of three conditions. The control solutions were either boiled for three minutes or exposed to neutralizing polyclonal antibodies to tissue plasminogen activator (rt-PA), or exposed to neutralizing polyclonal antibodies to urokinase plasminogen activator. Fibrinolytic activity was lost in the samples that were boiled or those that were exposed to polyclonal antibodies to rt-PA. On the other hand, the samples that were exposed to polyclonal antibodies to urokinase plasminogen activator retained activity. Activity for this part of the study was defined on the basis of the relative potency of rt-PA to urokinase. A loss of more than 10% of the original fibrinolytic activity of rt-PA is considered a clinically significant loss of potency [7].

### 3.3. Statistical Analysis

rt-PA measurements were expressed as Mean ± SEM and were compared using paired Student’s t-test. The inter-month variability was analyzed by one way-ANOVA.

## 4. Conclusions

The stability of frozen reconstituted rt-PA at −30 °C was investigated in this comprehensive study. These results, when compared with previous literature, revealed that such storage conditions do not negatively affect the stability of rt-PA for a maximum period of 3 months of storage while storing reconstituted rt-PA at −30 °C for longer durations resulted in significant loss in stability. It is also recommended, when feasible, that institutions should do their own rt-PA stability testing or use the shortest safe storage duration according to available literature.
